# Immunogenicity of a Bivalent Typhoid–Paratyphoid Conjugate Vaccine Against Diverse *Salmonella* Paratyphi A Isolates

**DOI:** 10.1093/infdis/jiag275

**Published:** 2026-06-03

**Authors:** Martina Carducci, Eleanna Sarakinou, Luisa Massai, Luca Rovetini, Mohammad AbdelGhany, Chiara Fineschi, Simona Nigro, Elisa Marchetti, Rita La Gaetana, Francesco Berlanda Scorza, Ashwani Kumar Arora, Valentino Conti, Omar Rossi, Simona Rondini

**Affiliations:** GSK Vaccines Institute for Global Health (GVGH) S.r.l., Siena, Italy; GSK Vaccines Institute for Global Health (GVGH) S.r.l., Siena, Italy; GSK Vaccines Institute for Global Health (GVGH) S.r.l., Siena, Italy; GSK Vaccines Institute for Global Health (GVGH) S.r.l., Siena, Italy; GSK Vaccines Institute for Global Health (GVGH) S.r.l., Siena, Italy; GSK Vaccines Institute for Global Health (GVGH) S.r.l., Siena, Italy; GSK Vaccines Institute for Global Health (GVGH) S.r.l., Siena, Italy; GSK Vaccines Institute for Global Health (GVGH) S.r.l., Siena, Italy; GSK Vaccines Institute for Global Health (GVGH) S.r.l., Siena, Italy; GSK Vaccines Institute for Global Health (GVGH) S.r.l., Siena, Italy; GSK Vaccines Institute for Global Health (GVGH) S.r.l., Siena, Italy; GSK Vaccines Institute for Global Health (GVGH) S.r.l., Siena, Italy; GSK Vaccines Institute for Global Health (GVGH) S.r.l., Siena, Italy; GSK Vaccines Institute for Global Health (GVGH) S.r.l., Siena, Italy

**Keywords:** enteric fever, *Salmonella* Paratyphi A, bivalent conjugate vaccine, mucosal immunity

## Abstract

**Background:**

In a first-in-human, phase 1, randomized controlled trial, a bivalent conjugate vaccine (Vi-CRM_197_  *+* O:2 CRM_197_) elicited robust immunoglobulin G (IgG) and bactericidal activity against vaccine-homologous *Salmonella* Paratyphi A strain. To further characterize the immune response, we assessed O:2 antigen-specific immunoglobulin A (IgA) and immunoglobulin M (IgM) responses as markers of mucosal immunity and early immune activation, and evaluated serum bactericidal activity against diverse *S* Paratyphi A isolates.

**Methods:**

The trial (NCT05613205) was conducted in 3 steps. For this exploratory analysis, 46 participants (mean age 27.2 years; 67.4% women) from Step 2 who received 2 full doses of Vi-CRM_197_  *+* O:2 CRM_197_ vaccine with and without aluminum hydroxide (alum) 169 days apart were considered. O:2-specific IgA/IgM responses and bactericidal antibodies were assessed. Geometric mean titers and concentrations and within-participant geometric mean ratios were calculated.

**Results:**

Both formulations induced strong anti-O:2 IgA/IgM responses and bactericidal activity, with higher responses in the nonadjuvanted group. At day 29 post–dose 1, IgA rose 74.14- and 16.65-fold (without vs with alum), remaining well above baseline through day 197. The nonadjuvanted formulation induced significantly higher IgA responses and bactericidal activity at day 29 (*P* < .05). Anti-O:2 IgM strongly correlated with bactericidal antibodies against homologous and heterologous strains.

**Conclusions:**

The vaccine effectively induced O:2-specific IgA, IgM, and bactericidal antibodies against diverse *S* Paratyphi A isolates, indicating potential broad protection.

**Clinical Trials Registration.** NCT05613205.

## Teaser

The bivalent combination of typhoid–paratyphoid conjugate vaccine (Vi-CRM_197_  *+* O:2 CRM_197_) induced strong IgA, IgM, and IgG responses and bactericidal activity against diverse *Salmonella* Paratyphi A strains, supporting its potential for comprehensive protection, especially for endemic regions.

Enteric fever, caused by *Salmonella* Typhi and Paratyphi, remains a major global health challenge, with an estimated 9.3 million cases and 107 500 deaths in 2021, including 2.17 million cases and 14 300 deaths due to *S* Paratyphi A [1, 2]. This burden is highly underestimated due to limited diagnostics, inadequate laboratory facilities, and widespread empirical antibiotic use [3, 4]. Geographically, enteric fever exhibits considerable variation, with South Asia accounting for approximately 62% of global cases, followed by sub-Saharan Africa (14%) and Southeast/East Asia and Oceania (12%) [2]. Although incidence is lower in developed countries, travelers from endemic regions remain at risk, with enteric fever being second only to malaria in severe, occasionally life-threatening infection [5–7]. Humans are the only carriers of these serovars, transmitting the infection primarily via contaminated food or water [5]. Disease severity ranges from mild fever and malaise to severe complications such as gastrointestinal bleeding, intestinal perforation, or encephalopathy [8]. Case fatality in endemic countries is estimated at 2.5%, rising to 4.5% in hospitalized patients, with probability of dying of 6.8% for antimicrobial-resistant strains [9]. Mortality is highest among children <5 years of age, accounting for approximately 40% of all fatalities [2, 10]. Additionally, 2%–5% of recovered patients become asymptomatic carriers, perpetuating transmission [8, 11]. While antibiotics remain the mainstay of treatment, their effectiveness is threatened by the emergence of extensively drug-resistant *Salmonella* strains [2, 12, 13]. Limited access to clean water and sanitation further complicates disease control [12]. Consequently, vaccination has gained increasing importance. A standalone *S* Paratyphi A vaccine is unlikely to be used, but a bivalent vaccine against *S* Typhi and *S* Paratyphi A would be a valuable public health tool. Currently, none of the available vaccines target *S* Paratyphi A [14]. Although *S* Typhi and *S* Paratyphi A cause clinically similar disease, they are genetically and phenotypically distinct; notably, the Vi polysaccharide capsule, a virulent factor of *S* Typhi, is absent in *S* Paratyphi A [15]. As a result, existing typhoid vaccines offer little or no cross-protection [16]. Instead, *S* Paratyphi A expresses the O:2 antigen, the serovar-specific, highly variable outer domain of the lipopolysaccharide. O:2 is a key virulence factor, facilitating adherence, colonization, and immune evasion, and is a primary vaccine target, with antibodies against O:2 believed to confer protective immunity [17–20].

A first-in-human phase 1 trial in healthy European adults showed that a low dose (5 µg of each antigen) and full dose (25 µg of each antigen) of Vi-CRM_197_ + O:2-CRM_197_ candidate vaccine, with or without aluminum hydroxide adjuvant (alum), elicited robust immunoglobulin G (IgG) responses to both antigens and high bactericidal activity against homologous *S* Paratyphi A strains and was well tolerated. While all vaccine formulations were immunogenic, the full dose without alum formulation demonstrated better immunogenicity, highlighting its potential for further clinical development [21]. However, the O-antigen is prone to structural heterogeneity driven by mechanisms such as lateral gene transfer, O-antigen conversion and phase variation, including fucosylation, glucosylation, acetylation, and alterations in O-antigen size [17, 18]. For example, in Nepal, approximately 10% of the *S* Paratyphi A clinical isolates showed structural variation [18]. Another study from the region revealed functional and phenotypic variations among isolates during outbreak [22]. Thus, an ideal vaccine would be one that can offer broad coverage, induce immunity against multiple strains, and remain effective considering continuous bacterial evolution. While the Vi-CRM_197_ + O:2-CRM_197_ vaccine elicited robust immune responses against homologous *S* Paratyphi A strain in the first-in-human study and against heterologous strains in preclinical studies, it was important to further investigate its ability to generate a robust immune response against heterologous strains in humans.

The objective of this exploratory analysis was to evaluate the immunogenicity of the full-dose Vi-CRM_197_ + O:2-CRM_197_ formulation with and without alum against heterologous strains, measured using serum bactericidal activity. Another objective was to assess immunoglobulin A (IgA) and immunoglobulin M (IgM) responses to support clinical development and enhance understanding of vaccine-induced immunity. IgA is essential for protecting mucosal surfaces, the primary site of infection, owing to their large surface area and constant microbial exposure [23]. It may also serve as a surrogate marker of protection, as evidenced by a study in which participants were immunized against *S* Typhi [24]. IgM is the first antibody secreted in response to infection, forming the initial line of defense [25].

## MATERIALS AND METHODS

### Study Design and Participants

This phase 1, observer-blind, controlled, self-contained, randomized, single-center, dose-escalation study was conducted at the Centre for Evaluation of Vaccination, University of Antwerp, Belgium (November 2022–April 2024) in accordance with international guidelines, including the Declaration of Helsinki, International Council for Harmonisation Good Clinical Practice guideline, and all applicable laws and regulations (ClinicalTrials.gov identifier NCT05613205). The primary and secondary endpoints of the study have been published [21]. The study consisted of 3 steps, in which 96 healthy European adults aged 18–50 years were enrolled. In Steps 1a and 1b, 18 participants were sequentially assigned and randomized (2:1) to receive either the low-dose vaccine (containing 5 µg of each antigen) without or with alum, or the control. In Step 2, 60 participants were randomized (2:2:1) to receive the full-dose vaccine (containing 25 µg of each antigen) without alum, the full-dose vaccine with alum, or a control vaccine. Participants received 2 doses of the candidate vaccine, administered 169 days apart. After the first interim analysis (day 28 post–dose 1), the study remained single-blind, with participants and investigator remaining blinded.

All participants from Step 2 of the study who were randomized to full-dose with alum (n = 24) and without alum (n = 22) were chosen for this exploratory analysis. As the analysis aimed to characterize immune responses among vaccinated participants, controls were excluded, and comparisons were made to baseline values. No additional assessments or samples collection were performed.

The panel of vaccine heterologous *S* Paratyphi A strains used in this study (02TY187, ED766, ATCC9150) was representative of overall genomic distribution and contained the most differing ones in terms of polysaccharide chain length, quantity, percentage of O-acetyl groups, and glucosylation content with emphasis on those exhibiting the most extreme O-antigen characteristics [18, 26, 27]. Characteristics of the heterologous and homologous strains are presented in Table 1.

**Table 1.** Clinical Isolates of *Salmonella* Paratyphi A Strains and Their O-Antigen Characteristics

Isolate^a^	OAg µg/OD	Size (kDa) HMW	MMW; Core	%OAc^b^	%Glc^b^
ED766	139	91.6	10.4; 2.7	100	79
02TY187	245	92.0	11.0; 2.5	37	95
ATCC9150	44	99.7	6.6; 2.6	17	57
ED199	219	86.1	0; 2.5	61	78

Source: Pinto et al [26].

Abbreviations: Glc, glucosylation; HMW, high molecular weight; kDa, kilodalton; MMW, medium molecular weight; OAc, O-acetylation; OAg, O-antigen; OD, optical density.

^a^In the published literature ED766, 02TY187, and ED199 are referred to as ED766, ED768, and NVGH308, respectively.

^b^%OAc and %Glc are expressed as molar ratio percentage with respect to repeating unit.

### Immunogenicity Assessments

The endpoints included the assessment of anti-O:2 IgA and IgM antibodies against the homologous *S* Paratyphi A strain measured by enzyme-linked immunosorbent assay (ELISA), for which standard curve was run in duplicate on each plate. The ELISA was performed using *S* Paratyphi A O-antigen as coating antigen. Results were expressed in ELISA units (EU)/mL with the lower limit of quantification of 14.7 EU/mL and 10.5 EU/mL, respectively. One EU is defined as the reciprocal of the dilution of the standard serum that gives an absorbance value (optical density measured at 405 nm minus that measured at 490 nm) equal to 1 in the assay.

In addition, the assessment of bactericidal antibodies against heterologous *S* Paratyphi A strains (02TY187, ED766, and ATCC9150), was performed using luminescence serum bactericidal assay [28–30]. Results were expressed as serum titers, defined as the serum dilutions yielding 50% inhibition of bacterial growth (IC_50_). The lower limits of quantification for bactericidal antibodies against *S* Paratyphi A ED766, 02TY187, and ATCC9150 were 5.7, 14.3, and 19.7 IC_50_, respectively [30]. Bactericidal antibodies against the homologous *S* Paratyphi A have been published [21].

### Statistical Analysis

The population for analysis comprised full-dose with and without alum participants in the per-protocol set (PPS), defined as all eligible participants who received each dose as per-protocol and had postvaccination immunogenicity data. Participants complied with dosing/blood-draw intervals, and had no intercurrent conditions affecting immunogenicity at each timepoint.

Geometric mean titers (GMTs) and within-participant geometric mean concentration/titer ratios (GMRs), also expressed as geometric mean fold rise (GMFR), of bactericidal antibodies against heterologous *S* Paratyphi A strains were calculated with corresponding 95% confidence intervals (CIs). Similarly, geometric mean concentrations (GMCs) and within-participant GMRs, reported as GMFR, of anti-O:2 IgA and IgM antibodies were calculated with their 95% CIs. The percentage of participants with ≥4-fold increase from baseline levels were calculated with their 95% CI, using the Clopper–Pearson method.

Both adjusted and unadjusted between-group ratios of GMCs and GMTs with 95% CIs were computed for full-dose with alum versus full-dose without alum. Unadjusted ratios were estimated using a linear model with results from the corresponding timepoint as the dependent variable and treatment group as the independent variable. Adjusted ratios were calculated from a linear mixed model with fixed effects for treatment, timepoint, treatment*timepoint interaction, baseline value, and a repeated timepoint effect within participant under an exchangeable covariance matrix, assuming unequal variance between groups. Given the exploratory nature of this analysis, all statistical comparisons were descriptive and no adjustment for multiplicity was applied.

Reverse cumulative distribution (RCD) curves were generated to illustrate the distribution of antibody concentrations by treatment group. Correlation analysis between anti-O:2 IgG, IgM, and IgA concentrations and bactericidal antibody titers against heterologous and homologous *S* Paratyphi A strains was performed for each timepoint and treatment using Pearson correlation coefficient based on log_10_-transformed data.

All statistical analyses were performed using SAS software version 9.4 (SAS Institute, Cary, North Carolina, USA).

## RESULTS

### Study Participants

All 46 participants who received the full-dose Vi-CRM_197_ + O:2-CRM_197_ vaccine were analyzed. Demographic and baseline characteristics were comparable across both groups (Table 2).

**Table 2.** Demographic and Baseline Characteristics of the Study Participants (Per-Protocol Set)

Characteristic	Full-Dose Without Alum (n = 22)	Full-Dose With Alum (n = 24)	Total (n = 46)
Age, y, mean ± SD	27.3 ± 9.6	27.1 ± 9.8	27.2 ± 9.6
Sex, No. (%)			
Female	14 (63.6)	17 (70.8)	31 (67.4)
Male	8 (36.4)	7 (29.2)	15 (32.6)
Weight, kg, mean ± SD	74.4 ± 12.5	71.3 ± 13.2	72.8 ± 12.8
BMI, kg/m^2^, mean ± SD	24.9 ± 3.7	23.5 ± 3.4	24.2 ± 3.6

Abbreviations: Alum, aluminum hydroxide; BMI, body mass index; SD, standard deviation.

### Immunogenicity

#### Anti-*S* Paratyphi A O:2 IgA and IgM

Overall, both the full-dose with and without alum formulations showed substantial increases in anti-O:2 IgA and IgM GMCs over baseline (Figure 1). The increase was greater in participants who received the full-dose without alum formulation compared to those who received full-dose with alum formulation, and remained well above baseline through day 197 (Table 3).

**Figure 1.**
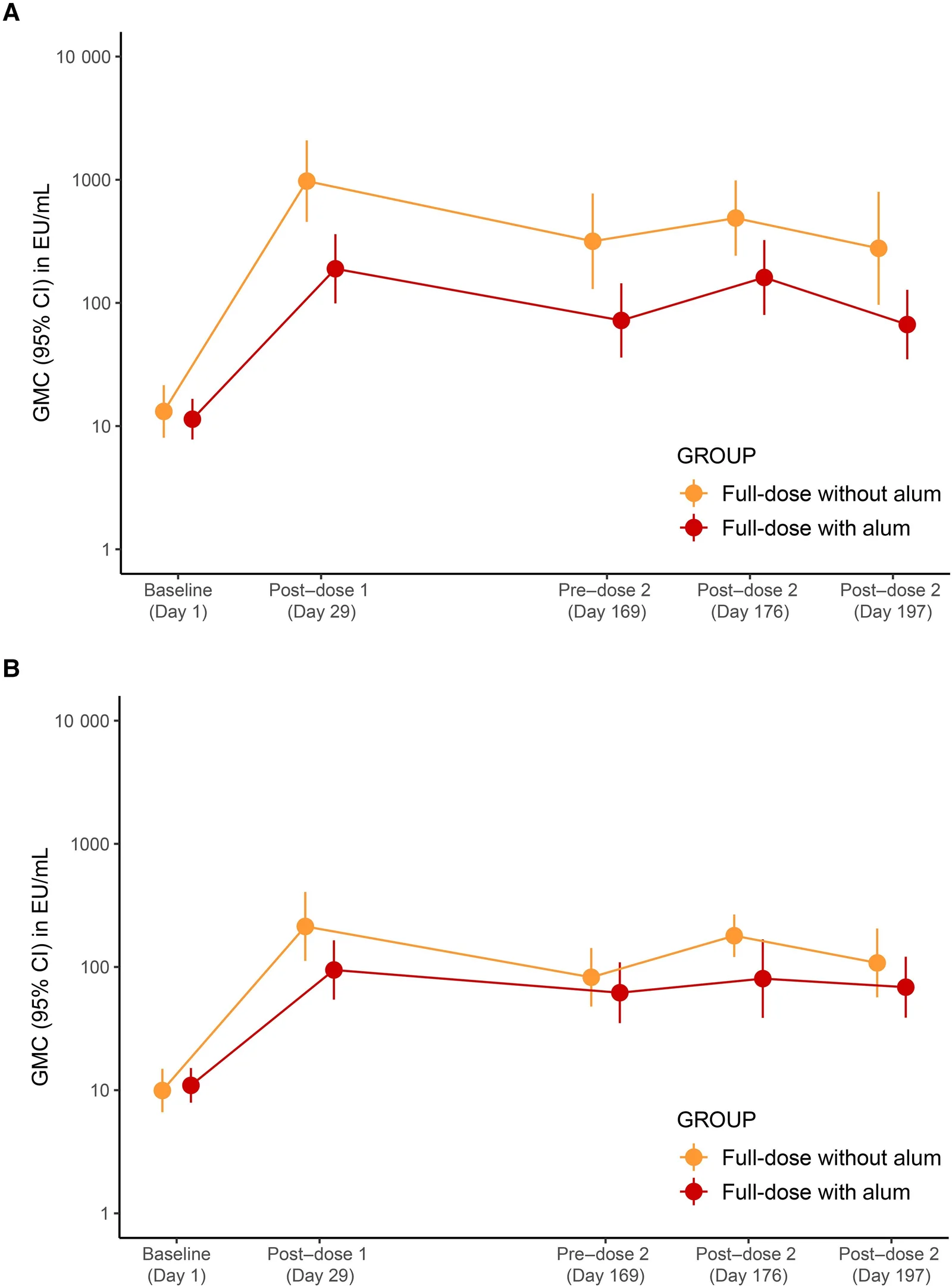
Anti-O:2 IgA (*A*) and IgM (*B*) antibody GMCs against *Salmonella* Paratyphi A homologous strain (per-protocol set). Abbreviations: Alum, aluminum hydroxide; CI, confidence interval; EU, enzyme-linked immunosorbent assay units; GMC, geometric mean concentration; IgA, immunoglobulin A; IgM, immunoglobulin M.

**Table 3.** Geometric Mean Concentrations, Geometric Mean Fold Rise, and Fold Increase for Anti-O:2 IgA and IgM Antibodies Against *Salmonella* Paratyphi A Homologous Strain^a^ (Per-Protocol Set)

Timepoint	Full-Dose Without Alum				Full-Dose With Alum			
	N	GMC (95% CI)	GMFR (95% CI)	Participants With at Least 4-Fold Increase From Baseline, % (95% CI)	N	GMC (95% CI)	GMFR (95% CI)	Participants With at Least 4-Fold Increase From Baseline, % (95% CI)
	Anti-O:2 IgA				Anti-O:2 IgA			
Day 1	21	13.15 (8.05–21.47)	…	…	24	11.37 (7.78–16.62)	…	…
Day 29	21	975.05 (455.03–2089.35)*	74.14 (39.90–137.78)	100 (83.9–100.0)	24	189.30 (99.21–361.23)	16.65 (9.10–30.47)	75.0 (53.3–90.2)
Day 169	21	316.57 (129.74–772.44)*	28.81 (13.30–62.42)	71.4 (47.8–88.7)	22	72.03 (36.00–144.14)	6.09 (3.21–11.55)	40.9 (20.7–63.6)
Day 176	18	488.31 (241.89–985.74)*	50.76 (26.61–96.83)	94.4 (72.7–99.9)	18	160.78 (79.86–323.71)	16.09 (7.78–33.29)	77.8 (52.4–93.6)
Day 197	16	277.46 (96.56–797.23)*	26.16 (10.57–64.74)	68.8 (41.3–89.0)	18	66.73 (34.89–127.65)	6.11 (3.47–10.74)	44.4 (21.5–69.2)
		Anti-O:2 IgM				Anti-O:2 IgM		
Day 1	21	9.93 (6.62–14.90)	**…**	…	24	10.93 (7.91–15.11)	…	…
Day 29	21	213.44 (112.17–406.14)	21.49 (10.65–43.38)	81.0 (58.1–94.6)	24	94.57 (54.34–164.56)	8.65 (4.99–14.99)	66.7 (44.7–84.4)
Day 169	21	82.48 (47.79–142.36)	8.31 (4.56–15.12)	66.7 (43.0–85.4)	22	61.81 (35.02–109.07)	5.92 (3.51–9.99)	59.1 (36.4–79.3)
Day 176	18	179.39 (120.72–266.57)	16.25 (8.96–29.46)	83.3 (58.6–96.4)	18	80.42 (38.63–167.42)	7.48 (3.86–14.51)	66.7 (41.0–86.7)
Day 197	16	107.92 (56.75–205.24)	11.35 (5.54–23.25)	75.0 (47.6–92.7)	18	68.56 (38.80–121.15)	6.30 (3.51–11.32)	55.6 (30.8–78.5)

Abbreviations: Alum, aluminum hydroxide; CI, confidence interval; GMC, geometric mean concentration; GMFR, geometric mean fold rise; IgA, immunoglobulin A; IgM, immunoglobulin M; N, number of samples tested.

^a^Homologous *S* Paratyphi A strain is identified as ED199.

^*^ *P* < .05 for unadjusted and adjusted between-group geometric mean ratios.

At day 29 post–dose 1, the GMFR for anti-O:2 IgA reached 74.14 in the full-dose without alum group versus 16.65 in the full-dose with alum group. The corresponding GMFRs at day 197 were 26.16- and 6.11-fold, respectively (Table 3).

Similarly, at day 29, the GMFR for anti-O:2 IgM reached 21.49 in the full-dose without alum group versus 8.65 in the full-dose with alum group, with corresponding values at day 197 of 11.35- and 6.30-fold, respectively (Table 3).

No boosting effect was observed post–dose 2, as indicated by the day 197 GMFRs relative to prevaccination values at day 169 for anti-O:2 IgA (full-dose without alum: 1.29 [95% CI, 1.08–1.54]; full-dose with alum: 1.28 [95% CI, 1.06–1.54]), and anti-O:2 IgM (full-dose without alum: 1.20 [95% CI, 1.07–1.35]; full-dose with alum: 1.11 [95% CI, 1.04–1.20]). The RCD curves of anti-O:2 IgA and IgM are presented in Supplementary Figure 1.

Consistent with these findings, most participants in both groups achieved at least a 4-fold increase from baseline in anti-O:2 IgA and IgM levels. At day 29, ≥4-fold increases in anti-O:2 IgA (100% vs 75.0%) and IgM (81.0% vs 66.7%) were higher in the full-dose without alum group than the full-dose with alum group. At day 197, ≥4-fold increases in anti-O:2 IgA (68.8% vs 44.4%) and IgM (75.0% vs 55.6%) were higher in the full-dose without alum group than the full-dose with alum group (Table 3).

In a pairwise comparison, the unadjusted GMRs of anti-O:2 IgA and IgM antibodies between the full-dose with and without alum groups were consistently <1 across all timepoints, indicating higher immunogenicity of the full-dose without alum formulation. The difference was statistically significant for anti-O:2 IgA antibodies at all timepoints (*P* < .05; Table 4), and a similar trend was observed between anti-O:2 IgM at day 29 post–dose 1 (*P* = .0515).

**Table 4.** Statistical Comparison of Immunogenicity Variables: Unadjusted and Adjusted Between-Group Ratios (Full-Dose With Alum Versus Full-Dose Without Alum, Per-Protocol Set)

Timepoint	Anti-O:2 IgA		Anti-O:2 IgM		Bactericidal Antibodies Against 02TY187		Bactericidal Antibodies Against ED766		Bactericidal Antibodies Against ATCC9150	
	GMR (95% CI)	*P* Value	GMR (95% CI)	*P* Value	GMR (95% CI)	*P* Value	GMR (95% CI)	*P* Value	GMR (95% CI)	*P* Value
Unadjusted ratios										
Day 29	0.19 (.07–.51)	.0013	0.44 (.20–1.01)	.0515	0.40 (.19–.84)	.0172	0.36 (.18–.72)	.0050	0.40 (.21–.78)	.0083
Day 169	0.23 (.08–.68)	.0090	0.75 (.35–1.61)	.4505	…		…		…	
Day 176	0.33 (.13–.86)	.0239	0.45 (.20–1.00)	.0501	…		…		…	
Day 197	0.24 (.08–.77)	.0176	0.64 (.28–1.44)	.2691	0.54 (.22–1.30)	.1617	0.41 (.18–.98)	.0446	0.56 (.27–1.16)	.1149
Adjusted ratios										
Day 29	0.19 (.08–.46)	.0004	0.47 (.22–1.01)	.0519	0.43 (.21–.87)	.0196	0.43 (.22–.81)	.0109	0.43 (.23–.83)	.0124
Day 169	0.21 (.09–.51)	.0009	0.77 (.36–1.66)	.4919	…		…		…	
Day 176	0.26 (.11–.63)	.0037	0.69 (.32–1.50)	.3386	…		…		…	
Day 197	0.23 (.09–.56)	.0018	0.73 (.34–1.60)	.4297	0.57 (.27–1.20)	.1337	0.43 (.22–.85)	.0170	0.53 (.27–1.03)	.0613

Unadjusted measurements are based on a linear model for a visit with fixed effect for treatment. Adjusted measurements are based on a linear mixed model with fixed effect for treatment, timepoint, baseline value, treatment*timepoint, and a repeated timepoint effect within a participant under an exchangeable covariance matrix. Model assumes unequal variances between treatment groups.

Abbreviations: Alum, aluminum hydroxide; CI, confidence interval; GMR, geometric mean concentration/titer ratio; IgA, immunoglobulin A; IgM, immunoglobulin M.

### Serum Bactericidal Activity

The full-dose with alum and full-dose without alum formulations substantially increased GMTs of bactericidal antibodies against all heterologous *S* Paratyphi A strains at day 29, which remained above baseline through day 197 (Figure 2). The second dose did not further boost the response, as indicated by GMT levels at day 197. Bactericidal antibodies titers post–dose 1 and 2 were higher in the full-dose without alum group compared with the full-dose with alum group (Table 5). In addition, descriptive comparison of serum bactericidal antibody titers against homologous and heterologous *S* Paratyphi A strains is presented in Supplementary Figure 2.

**Figure 2.**
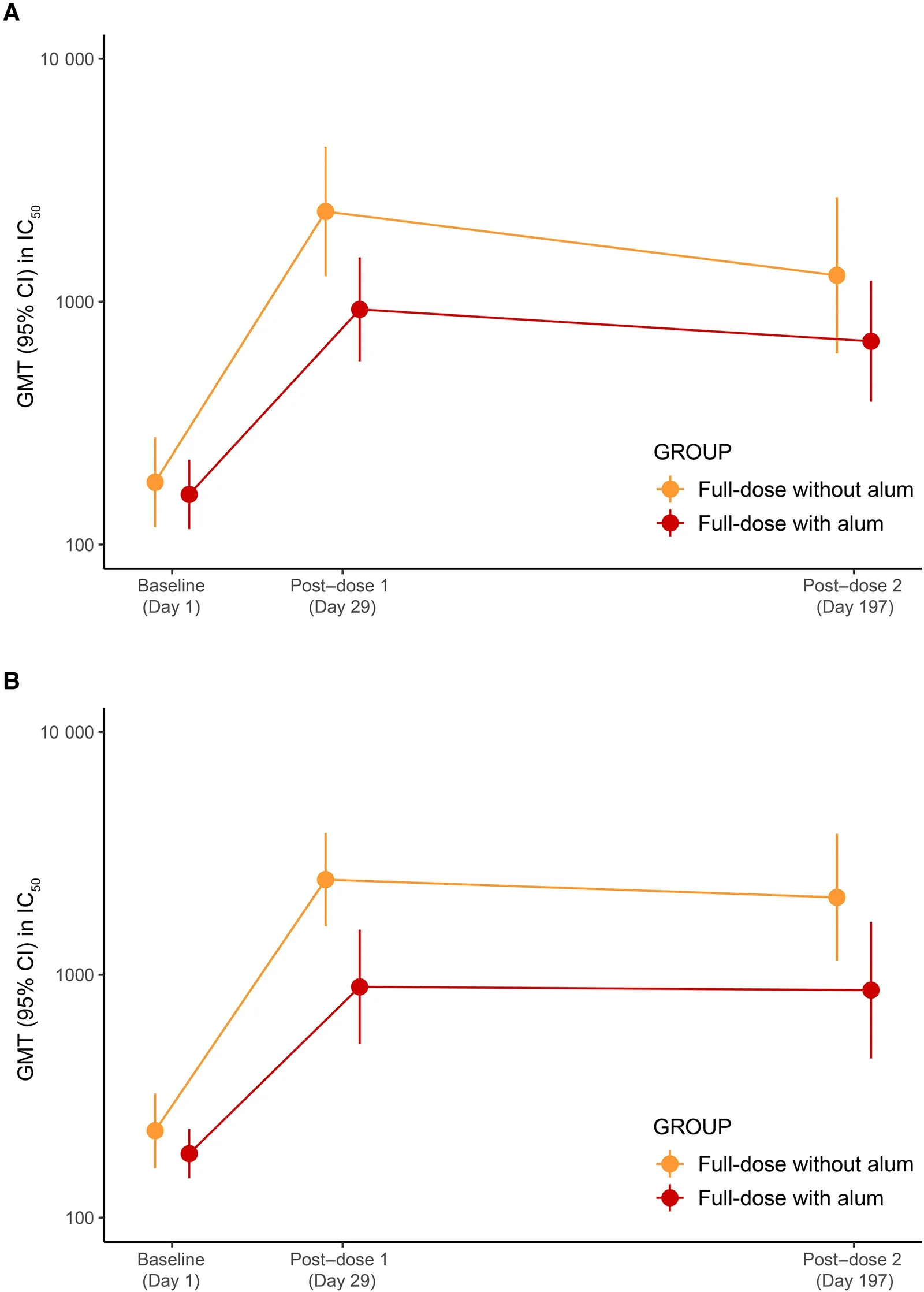

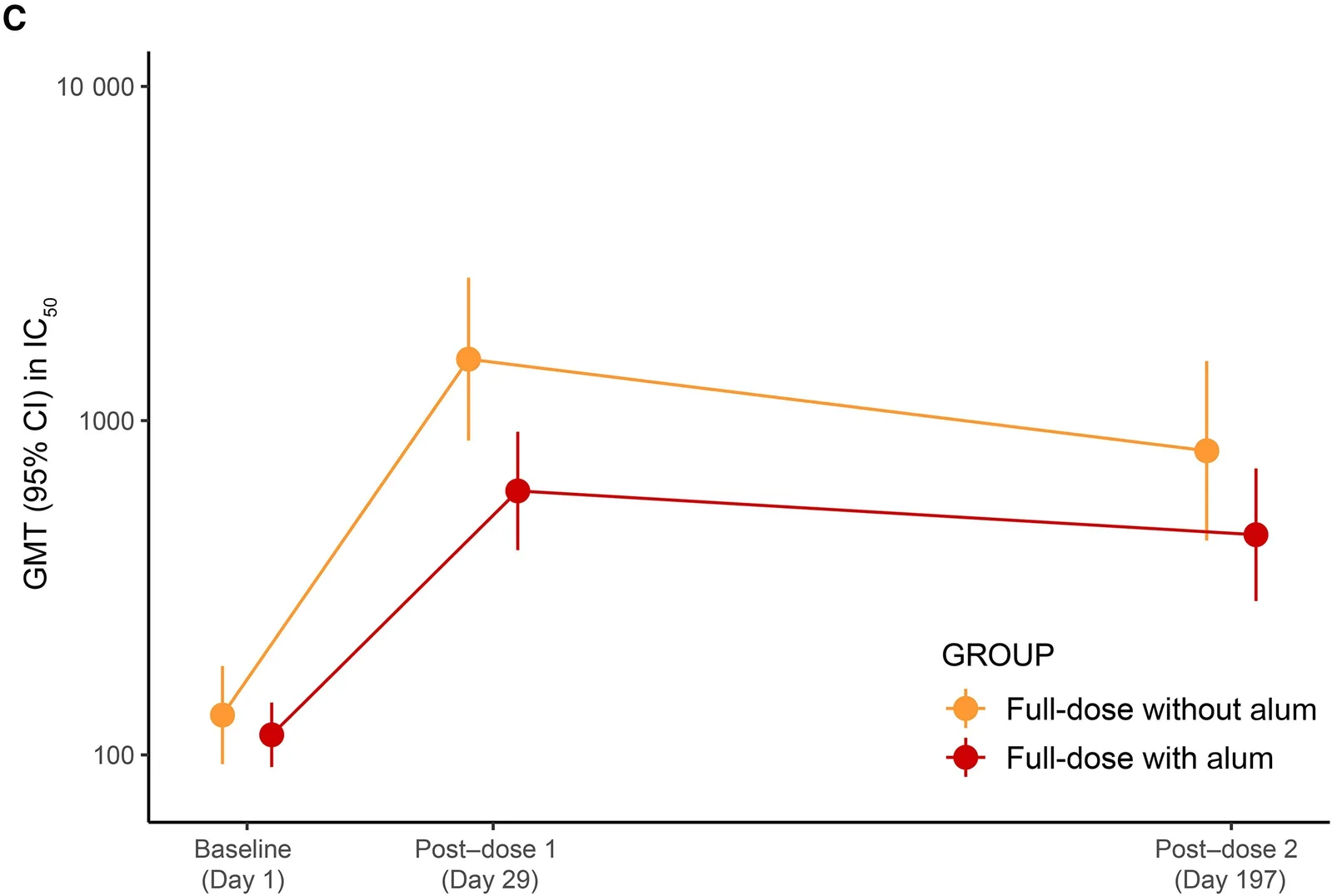
Bactericidal antibody GMTs against *Salmonella* Paratyphi A heterologous strains (per-protocol set): 02TY187 (*A*), ED766 (*B*), and ATCC9150 (*C*). Abbreviations: Alum, aluminum hydroxide; CI, confidence interval; GMT, geometric mean titers; IC_50_, 50% inhibition of bacterial growth.

**Table 5.** Geometric Mean Titers, Geometric Mean Fold Rises, and Fold Increase for Serum Bactericidal Antibodies Against *Salmonella* Paratyphi A Heterologous Strains (Per-Protocol Set)

Timepoint	Full-Dose Without Alum				Full-Dose With Alum			
	N	02TY187	ED766	ATCC9150	N	02TY187	ED766	ATCC9150
	GMT (95% CI)				GMT (95% CI)			
Day 1	21	180.62 (118.12–276.21)	228.16 (160.34–324.66)	131.61 (93.94–184.39)	24	160.86 (115.83–223.38)	183.52 (145.13–232.07)	114.89 (92.03–143.42)
Day 29	21	2350.31 (1273.22–4338.60)*	2465.36 (1585.42–3833.68)*	1527.65 (872.12–2675.90)*	24	929.52 (568.21–1520.57)	891.97 (518.46–1534.56)	616.29 (409.97–926.46)
Day 197	16	1283.31 (611.84–2691.69)	2083.87 (1140.66–3807.03)*	812.56 (437.84–1507.99)	18	687.31 (387.76–1218.27)	864.79 (452.78–1651.71)	455.73 (288.79–719.17)
		GMFR (95% CI)				GMFR (95% CI)		
Day 29	21	13.01 (6.93–24.44)	10.81 (6.68–17.48)	11.61 (6.46–20.85)	24	5.78 (3.74–8.94)	4.86 (3.11–7.59)	5.36 (3.63–7.94)
Day 197	16	7.61 (3.64–15.91)	10.18 (5.55–18.65)	6.86 (3.66–12.87)	18	4.62 (2.78–7.68)	4.80 (2.80–8.23)	4.12 (2.60–6.53)
		Participants With at Least 4-Fold Increase From Baseline, % (95% CI)				Participants With at Least 4-Fold Increase From Baseline, % (95% CI)		
Day 29	21	90.5 (69.6–98.8)	81.0 (58.1–94.6)	81.0 (58.1–94.6)	24	58.3 (36.6–77.9)	54.2 (32.8–74.4)	54.2 (32.8–74.4)
Day 197	16	62.5 (35.4–84.8)	81.3 (54.4–96.0)	62.5 (35.4–84.8)	18	55.6 (30.8–78.5)	55.6 (30.8–78.5)	50.0 (26.0–74.0)

Abbreviations: Alum, aluminum hydroxide; CI, confidence interval; GMFR, geometric mean fold rise; GMT, geometric mean titer; N, number of samples tested.

^*^ *P* < .05 for unadjusted and adjusted between-group geometric mean ratios.

At day 29, 81.0%–90.5% of participants in the full-dose without alum group and 54.2%–58.3% in the full-dose with alum group achieved at least 4-fold increase in bactericidal antibodies against heterologous *S* Paratyphi A strains. Similarly, at day 197, 62.5%–81.3% of participants in the full-dose without alum group and 50.0%–55.6% in the full-dose with alum group continued to maintain bactericidal antibodies levels at least 4-fold above baseline (Table 5). The RCD curves of bactericidal antibodies titers against heterologous *S* Paratyphi A strains are shown in Supplementary Figure 3.

In a pairwise comparison, the unadjusted GMRs for bactericidal antibodies against *S* Paratyphi A strains (02TY187, ED766, and ATCC9150) between the full-dose without alum and full-dose with alum groups were <1 at all timepoints, indicating that full-dose without alum was more immunogenic and elicited higher bactericidal activity. The difference was statistically significant for all 3 strains at day 29 (*P* < .05), but not at day 197 for 02TY187 and ATCC9150 (*P* > .05). The pairwise comparison of adjusted GMRs for bactericidal antibodies against heterologous *S* Paratyphi A strains between the full-dose without and with alum groups showed a similar trend (Table 4).

### Correlation Analyses

A moderate-to-strong correlation between anti-O:2 IgM concentration and bactericidal antibody titers against heterologous and homologous *S* Paratyphi A strains was observed (Supplementary Table 1), with correlation coefficients of 0.680–0.764 on day 29 and 0.738–0.812 on day 197 (Supplementary Figure 4).

No correlation was observed between anti-O:2 IgG antibody concentrations and bactericidal antibodies against heterologous (correlation coefficient: −0.321 to 0.283) and homologous *S* Paratyphi A strain at day 29 and day 197 (correlation coefficient: 0.012 to −0.636, respectively) (Supplementary Table 2).

## DISCUSSION

The persistent burden of enteric fever and rising antimicrobial resistance underscore the need for a bivalent *S* Typhi/*S* Paratyphi A vaccine, establishing them as a public health priority, particularly in Asia [31]. In a phase 1 trial, the Vi-CRM_197_ + O:2-CRM_197_ vaccine elicited strong anti-O:2 IgG and serum bactericidal responses against homologous *S* Paratyphi A in healthy European adults [21]. As part of the tertiary objective, we assessed anti-O:2 IgA and IgM levels, which are key for mucosal protection and early immunity, respectively. We also evaluated the vaccine's ability to elicit bactericidal activity against vaccine heterologous *S* Paratyphi A strains representative of circulating lineages and with substantial O-antigen features, as differences in O-antigen characteristics may reduce antibody binding and potentially limit vaccine recognition or coverage [18, 26, 32].

Overall, both the full-dose with and without alum formulations induced substantial increases in anti-O:2 IgA and IgM GMCs over baseline, with higher responses in the full-dose without alum group sustained through day 197. The markedly higher GMFR with the full-dose without alum compared with full-dose with alum in anti-O:2 IgA (74.14-fold vs 16.65-fold) and IgM (21.49-fold vs 8.65-fold) suggests that the nonadjuvanted formulation elicits a stronger and potentially more protective immune response. The observed rise in IgA antibodies is particularly relevant, as mucosal IgA plays key roles in controlling infections. It prevents bacterial adhesion to the intestinal lining by neutralizing pathogens and blocking infection. Evidence from controlled human infection models of *S* Typhi show that protected individuals exhibited 2.0- to 3.5-fold higher Vi-specific IgA compared with individuals diagnosed with typhoid fever, which correlated with protection against challenge [24]. While mucosal protection is primarily mediated by secretory IgA, elevated serum IgA levels are suggestive of vaccine-induced responses, and may serve as surrogate indicators of host immunity [33]. Beyond its mucosal role, serum IgA may contribute to systemic antibacterial activity via FcαRI-mediated myeloid cell activation (eg, opsonophagocytosis), though the magnitude and relevance of these effects remain to be fully established [34]. In contrast, IgM antibodies represent the first line of humoral defense following an antigen exposure and are believed to play an important role in activation of the complement system, essential for efficient clearance of pathogens [25, 35]. High IgM levels may be particularly important for controlling early stages of infection and limiting its spread. Moreover, the high anti-O:2 IgM concentrations, along with the moderate-to-strong correlations observed between anti-O:2 IgM and bactericidal antibodies against both homologous and heterologous *S* Paratyphi A strains, suggest that IgM antibodies contribute directly to complement-mediated killing. This aligns with the well-established ability of pentameric IgM to efficiently fix complement [36]. Furthermore, though the second dose did not boost the IgA and IgM immune response, an increase of approximately 1.1- to 1.3-fold compared to pre–dose 2 levels suggests that the first dose adequately elicited the antigen-specific humoral response. This is consistent with the observed postdose antibody response, where following the first dose, 100% and 81.0% of the participants in the full-dose without alum group achieved at least 4-fold increase in IgA and IgM antibodies, respectively. Similarly, for anti-O:2 IgG, the second dose did not boost responses [21].

Importantly, the rise in IgM and IgA levels was complemented by a robust IgG response, with a 162.61-fold increase in antibodies and 100% of participants in the full-dose without alum group achieving at least a 4-fold rise [21]. While IgA, IgG, and IgM levels provide evidence of vaccine immunogenicity, bactericidal activity reflects their functional capacity for complement-dependent bacterial killing and is crucial for understanding protective potential [37]. We have previously shown that 1 month post–dose 1 of the Vi-CRM_197_ + O:2-CRM_197_ vaccine, bactericidal antibodies against *S* Paratyphi A increased substantially and remained above baseline levels until the second dose administration [21]. However, to account for differences in O-antigen characteristics, we tested *S* Paratyphi A clinical strains representative of O-antigen modifications that could be encountered, to assess the breadth of vaccine-induced neutralizing antibodies. Both full-dose formulations elicited robust bactericidal activity against heterologous *S* Paratyphi A strains (approximately11- to 13-fold for full-dose without alum; approximately5- to 6-fold for full-dose with alum) post–dose 1, with the nonadjuvanted formulation showing significantly higher functional response across all strains (*P* < .05). The Vi-CRM_197_ + O:2-CRM_197_ vaccine was able to generate bactericidal antibodies against heterologous vaccine *S* Paratyphi A strains (02TY187, ED766, and ATCC9150), indicating cross-reactive antibody activity. Furthermore, in rabbits, the Paratyphi A component of the vaccine induced functional antibodies capable of effectively binding to and mediating bactericidal activity against diverse *S* Paratyphi A isolates, regardless of O:2 structural differences [26].

In conclusion, the bivalent conjugate Vi-CRM_197_ + O:2-CRM_197_ candidate vaccine, administered with or without adjuvant, elicited robust immune responses against both homologous and heterologous *S* Paratyphi A strains, irrespective of the O-antigen structural variations. The vaccine induced strong IgA and IgM responses, which are critical for mucosal defense, complement activation, and early pathogen clearance, and these were complemented by a robust IgG response, together highlighting a potential for comprehensive and sustained coverage. Furthermore, it also demonstrated the ability to induce bactericidal activity across diverse *S* Paratyphi A strains, underscoring the potential for broad coverage. Given that no paratyphoid vaccines are currently licensed, the development of a bivalent vaccine with the capacity to protect against both typhoid and paratyphoid fever would represent a major advance, particularly for low- and middle-income countries and for travelers to endemic regions.

The limitations of this study include its exploratory design and the small number of participants, primarily from European regions, which may not fully reflect immune responses in target infant populations from endemic settings. Also, the lower immunogenicity observed with the adjuvanted formulation remains unclear; although similar findings have been reported [38], this study was not designed to assess formulation-related mechanisms, including potential effects of adsorption to alum on conjugate stability. Moreover, as the correlate of protection has not yet been established, evaluating additional antibody characteristics such as functionality assessment, FcR binding, IgG subclass analysis, and avidity may provide a more comprehensive understanding of immunity, which can eventually be extended to future studies. Altogether, these findings provide important insights into vaccine-induced immunity and support future research, including clinical development of the single full-dose nonadjuvanted formulation. Larger studies in endemic regions are necessary to validate and extend these observations in the target populations.

## Supplementary Material

jiag275_Supplementary_Data

## Supplementary Data

Supplementary materials are available at *The Journal of Infectious Diseases* online (http://jid.oxfordjournals.org/). Supplementary materials consist of data provided by the author that are published to benefit the reader. The posted materials are not copyedited. The contents of all supplementary data are the sole responsibility of the authors. Questions or messages regarding errors should be addressed to the author.
