# Supplementary material for: Immunogenicity of a Bivalent Typhoid–Paratyphoid Conjugate Vaccine Against Diverse Salmonella Paratyphi A Isolates
Source: J Infect Dis. 2026 Jun 3;234(4):e786–96. doi: 10.1093/infdis/jiag275 (PMC13668349; doi:10.1093/infdis/jiag275)
Supplement: Supplementary file 1 [file jiag275_supplementary_data.docx]

**Supplement**

**Supplementary Figure S1: Reverse cumulative distribution curves of anti-O:2 IgA and IgM concentrations against homologous *S.* Paratyphi A strain* by treatment group (PPS)**

**A: Anti-O:2 IgA**

**B: Anti-O:2 IgM**

Alum, aluminum hydroxide; IgA, immunoglobulin A; IgM, immunoglobulin M; N, number of participants with available results per timepoint; PPS, Per-Protocol Set.

The lower limit of quantification for anti-O:2 IgA and IgM as detected by enzyme-linked immunosorbent assay was 14.7 EISA units/mL and 10.5 ELISA units/mL, respectively (represented by the vertical line).

*Homologous *S.* Paratyphi A strain is identified as ED199

**Supplementary Figure S2: Comparison of serum bactericidal antibodies GMTs against homologous* and heterologous *S.* Paratyphi A** **strains (PPS)**

**A: Full-dose with Alum**

IC_50_

**B: Full-dose without Alum**

IC_50_

Alum, Aluminum hydroxide; IC_50,_ 50.0% inhibition of bacterial growth; GMT, Geometric mean titers; PPS, Per-Protocol Set

*Homologous *S.* Paratyphi A strain is identified as ED199

**Supplementary Figure S3: Reverse cumulative distribution curves of bactericidal antibodies against heterologous *S.* Paratyphi A strains from SBA by treatment group (PPS)**

**A: 02TY187**

**B: ED766**

**C: ATCC9150**

Alum, aluminum hydroxide; N, number of participants with available results per timepoint; PPS, Per-Protocol Set; SBA, serum bactericidal assay

The lower limit of quantification for serum bactericidal assay for *S.* Paratyphi A 02TY187, ED766 and ATCC9150 was 14.3 IC_50_, 5.7 IC_50_ (50.0% inhibition of bacterial growth), and 19.7 IC_50_ (represented by the vertical line).

**Supplementary Figure S4: Correlation between anti-O:2 IgM and bactericidal antibodies against homologous and heterologous *S.* Paratyphi A strains (PPS)**

**A: Day 29 post-dose 1**

**ED199*	02TY187**

SBA log10 Bactericidal Antibodies (IC_50_)

| **ED766** | **ATCC9150** |
| --- | --- |

SBA log10 Bactericidal Antibodies (IC_50_)

**B: Day 197**

| **ED199*** | **02TY187** |
| --- | --- |
|  |  |
| **ED766** | **ATCC9150** |
|   ELISA log10 anti-O:2 IgM (EU/mL) |   ELISA log10 anti-O:2 IgM (EU/mL) |

SBA log10 Bactericidal Antibodies (IC_50_)

SBA log10 Bactericidal Antibodies (IC_50_)

ELISA, enzyme-linked immunosorbent assay; EU, ELISA units; IC_50,_ 50.0% inhibition of bacterial growth; IgM, immunoglobulin M; log10, logarithm base 10; N, number samples tested; PPS, Per-Protocol Set; SBA, serum bactericidal assay

* Homologous *S.* Paratyphi A strain

**Supplementary Table S1: Correlation between anti-O:2 IgM and bactericidal antibodies against *S.* Paratyphi A strains (PPS)**

| **Time point** | **Full-dose without Alum**  **r** | | | | | **Full-dose with Alum**  **r** | | | | | |
| --- | --- | --- | --- | --- | --- | --- | --- | --- | --- | --- | --- |
|  | **N** | **02TY187** | **ED766** | **ATCC9150** | **ED199*** | **N** | **02TY187** | **ED766** | | **ATCC9150** | **ED199*** |
| **D1** | 21 | 0.501 | 0.517 | 0.569 | 0.387 | 24 | 0.405 | 0.349 | | 0.393 | 0.295 |
| **D29** | 21 | 0.616 | 0.561 | 0.631 | 0.654 | 24 | 0.679 | 0.716 | | 0.712 | 0.818 |
| **D169** | 21 |  |  |  | 0.682 | 22 |  |  | |  | 0.834 |
| **D176** | **18** |  |  |  | 0.715 | 18 |  | |  |  | 0.885 |
| **D197** | **16** | 0.840 | 0.691 | 0.811 | 0.852 | 18 | 0.718 | | 0.765 | 0.694 | 0.764 |
| **All Visits** | 58 | 0.835 | 0.823 | 0.851 | 0.821^a^ | 66 | 0.786 | 0.797 | | 0.814 | 0.835^b^ |

Alum, aluminum hydroxide; D, day; IgM, immunoglobulin M; N, number of samples tested; PPS, Per-Protocol Set; r, Pearson correlation coefficient.

^a^N=97; ^b^N=106

Pearson correlation coefficient on log10-transformed value is presented.

*Homologous *S.* Paratyphi A strain

**Supplementary Table S2: Correlation** **between anti-O:2 IgG and bactericidal antibodies against *S.* Paratyphi A strains (PPS)**

| **Time point** | **Full-dose without Alum**  **r** | | | | | **Full-dose with Alum**  **r** | | | | | **Total**  **r** | | |
| --- | --- | --- | --- | --- | --- | --- | --- | --- | --- | --- | --- | --- | --- |
|  | **N** | **02TY187** | **ED766** | **ATCC9150** | **ED199*** | **N** | **02TY187** | **ED766** | **ATCC9150** | **ED199*** | **02TY187** | **ED766** | **ATCC9150** |
| **D1** | 21 | -0.002 | 0.141 | 0.129 | 0.110 | 24 | -0.247 | -0.317 | -0.285 | -0.448 | -0.135 | -0.091 | -0.080 |
| **D29** | 21 | -0.156 | 0.000 | 0.076 | 0.050 | 24 | 0.080 | -0.037 | 0.169 | 0.012 | 0.143 | 0.178 | 0.283 |
| **D169** | 21 |  |  |  | -0.376 | 22 |  |  |  | -0.148 |  |  |  |
| **D176** | 18 |  |  |  | -0.443 | 18 |  |  |  | 0.007 |  |  |  |
| **D197** | 16 | -0.624 | -0.758 | -0.481 | -0.636 | 18 | -0.196 | -0.165 | -0.276 | -0.147 | -0.321 | -0.281 | -0.266 |
| **All visits** | 58 | 0.480 | 0.564 | 0.582 | 0.401^a^ | 66 | 0.388 | 0.334 | 0.457 | 0.264^b^ | 0.461 | 0.483 | 0.549 |

Alum, aluminum hydroxide; D, day; N, number of samples tested; PPS, Per-Protocol Set; r, Pearson correlation coefficient.

Pearson correlation coefficient on log10-transformed value is presented.

^a^N=97; ^b^N=106

*Homologous *S.* Paratyphi A strain
